# Whose Jurisdiction Is Home Contamination? Para-Occupational ‘Take-Home’ Herbicide Residue Exposure Risks among Forestry Workers’ Families in South Africa

**DOI:** 10.3390/ijerph181910341

**Published:** 2021-09-30

**Authors:** Bonolo Anita Pududu, Hanna-Andrea Rother

**Affiliations:** Division of Environmental Health, School of Public Health and Family Medicine, University of Cape Town, Observatory, Cape Town 7925, South Africa; pududuba@yahoo.com

**Keywords:** para-occupational ‘take-home’ exposure, pesticides, herbicides, personal protective equipment (PPE), forestry workers, chemical legislation, South Africa

## Abstract

Para-occupational “take-home” exposure risks among forestry workers and their families in low-and middle-income countries (LMICs) have not been well characterized. This is a concern because research shows an association between chronic low-dose herbicide exposure and adverse health effects. This study explored take-home herbicide residue exposure risks among forestry workers in the Western Cape, South Africa, through the community-based participatory research approach of photovoice. A key finding of the study was the absence of provisions related to take-home exposure in the national legislation and workplace policies, which largely contributed to poor adherence to risk reduction practices at worksites, in addition to workers transporting residues to their homes. This study demonstrated evidence of the key omissions regarding take-home exposure at the policy level (e.g., recommendations for employers to reduce take-home risks among employees, and training of workers and their families on take-home exposure) and take-home herbicide residue exposure among worker’s families, including children.

## 1. Introduction

Herbicides are a group of pesticides used extensively to eradicate and manage invasive alien plant species in South Africa. Exposure to herbicide residues, however, may be harmful to non-target organisms such as humans who work with, or come into contact with, residues [1,2,3]. This includes workers’ families who may be unintentionally exposed to residues at home through the para-occupational “take-home” exposure pathway. Evidence increasingly shows the association between herbicide exposure and adverse health effects [2,4,5,6,7,8]. This represents a significant public health problem, particularly in low- and middle-income countries (LMICs), where limited evidence exists on the incidence of occupational and, to an even lesser extent, non-occupational poisoning. Exposure risks in LMICs are a result of several different factors, which include: weak legislation and policies that regulate pesticide use, inadequate training, poor access to information and the means to comprehend this information, and minimal use of and access to personal protective equipment (PPE) ([9,10,11]. Research on take-home exposure risks among agricultural populations in high-income countries (HICs) is extensive and well documented. Given this existing evidence for agricultural populations, it is plausible that forestry workers, and their families, experience similar risks. However, the extent of take-home herbicide residue exposure risks among forestry workers and their families in LMICs is not known. 

### 1.1. Para-Occupational Take-Home Exposure 

Para-occupational take-home exposure has been defined as indirect exposure which occurs when workers unintentionally transport pesticide residues on their skin, hair, clothing, shoes, and vehicles from the workplace into their homes [12,13,14,15,16,17,18,19,20,21]. Concern for this exposure pathway was initially raised in 1995 by the Centers for Disease Control National Institute for Occupational Safety and Health (NIOSH) study, which reported that workers’ homes in 28 countries and 36 states in the United States (US) were contaminated with workplace chemicals, including pesticides [22,23]. Subsequent research conducted in HICs confirmed that pesticides, such as herbicides, accumulate in workers’ homes, potentially exposing their families to residues and the associated health risks [14,15,18,20,21,24,25,26,27]. A study among farmworkers in North Carolina, US, showed the persistence of pesticides in the indoor environment as concentrations of occupational pesticides no longer registered for use in the US were identified in their homes [28]. This suggests that there are difficulties in effectively decontaminating workers’ homes once residues have entered the indoor environment. Furthermore, it confirms that take-home exposure remains a risk for workers and their families. Children have been observed to have increased health risks of exposure compared to adults due to their high rates of metabolism, developing immune systems, and distinctly different behaviours (e.g., hand to mouth practices, playing on the floor) [12,14,20,21,27,29]. 

### 1.2. Personal Protective Equipment as a Source of Take-Home Exposure 

Although personal protective equipment (PPE) is an important measure to reduce the risk of pesticide exposure at work, it has been shown to be a source of take-home residues [30,31]. Workers transport workplace residues on their PPE (e.g., work clothes, work boots) into their homes, contaminating surfaces used by other household members [30,32,33,34]. Curwin et al. (2005) found evidence of herbicide residues (i.e., atrazine, metolachlor, glyphosate, and 2,4-D) being tracked into the homes of farmworkers in Iowa, US, on their clothing and boots. The study noted that the entrance ways, change areas, and laundry rooms in farm homes had higher concentrations of herbicide residues compared to other rooms in their homes [35]. Another study by Curl et al. (2002) in Yakima Valley, Washington, observed a strong association between azinphosmethyl concentrations detected in dust found in the homes and work vehicles of farmworkers. The concern is the potential health effects linked to these take-home exposures [14].

### 1.3. Herbicide Exposure and Chronic Health Effects

Long-term, low-dose exposure to pesticides has been associated with chronic health effects; however, the extent and severity of effects are not widely known in LMICs [16,36,37,38]. Exposure, specifically to herbicide residues, has been associated with non-Hodgkin lymphoma, Parkinson’s disease, asthma, type 2 diabetes, end stage renal disease, increased risk of anxiety and depression resulting in suicide, spontaneous miscarriage, and increased infant deaths, in addition to bladder, colon, and breast cancers [2,4,5,6,7,8]. Studies have further reported adverse health effects in children resulting from parental herbicide exposure; these include: leukemia, increased risk of childhood brain defects, adverse neurodevelopmental and neurobehavioral outcomes including attention deficit/hyperactivity disorder (ADHD), cognitive impairment, birth defects (e.g., orofacial clefts), asthma, and endocrine mimicking effects [2,6,17]. There is limited evidence which ascertains the extent to which these health effects can be attributed to take-home exposures. Only one study conducted in Iowa and North Carolina, US, examined the health effects of take-home exposure among a group of herbicide applicators and their female spouses, who were followed over a period of five years. The study found that long-term exposure to paraquat and butylate was associated with end-stage renal disease among female spouses who had never had direct contact with these herbicides [5]. This suggests that although the levels of exposure among workers’ families tend to be indirect and somewhat less compared to workers, families remain at risk of developing adverse health effects from these indirect exposures in the long term. The research presented in this article, therefore, documented the hygiene and other related practices (e.g., care and maintenance of PPE) of forestry workers contracted under the South African Working for Water (WfW) program to identify potential take-home herbicide residue exposure risks among their families. The WfW program is a national government-wide initiative which extensively uses herbicides for the removal of alien invasive plant species and has a policy of hiring unemployed members of marginalized and poor communities.

## 2. Materials and Methods

This qualitative study was part of a larger research project that explored WfW forestry workers’ herbicide exposure risks. Previous studies under the parent study focused on investigating factors related to workers’ occupational herbicide exposure risks [9,39]; however, none of these studies explored non-occupational exposure risks. 

### 2.1. Research Setting

**Figure 1:**
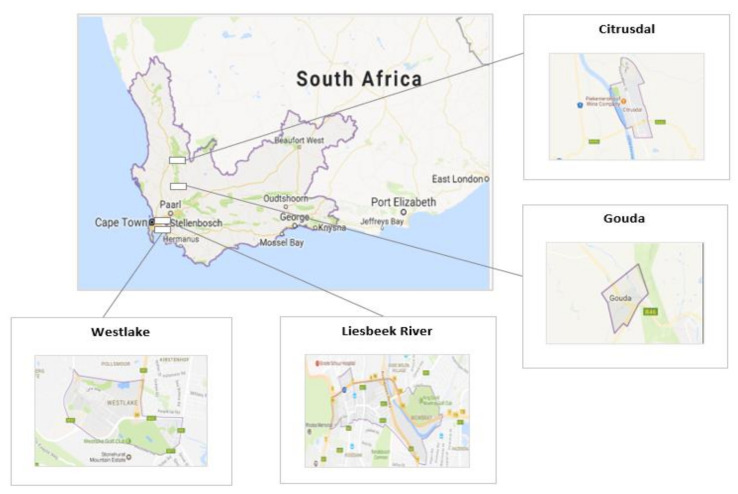
Location of the study sites in the Western Cape Province, South Africa.

This study was conducted at four sites in the Western Cape Province of South Africa (SA), namely: Gouda, Citrusdal, Liesbeek Rivers, and Westlake (Figure 1). The Western Cape is in the south-western part of SA, and is the fourth largest province in terms of land area (129,449 square kilometers) and has the fourth largest population (5.82 million people) in the country [40,41]. The province is rich in biological diversity; however, the increasing presence of invasive alien plant species poses a threat to both terrestrial and freshwater ecosystems [41,42]. In response, the WfW program was introduced to remove, control, and manage invasive alien plant species through mechanical and chemical control methods (e.g., herbicides), when deemed necessary, to restore indigenous low water consuming vegetation [43]. The estimated volume of herbicide use per hectare is as follows: medium infestation is 75%, sparse infestation is 50%, scattered infestation is 25%, very scattered infestation is 10%, and occasional infestation is 1%.

### 2.2. Study Population

The study population comprised of 37 WfW forestry workers from the four research sites, of which 65% of the workers were female. More than half of the study participants spoke in local languages other than English (i.e., Afrikaans—32% and isiXhosa—22%) (Table 1). Participants were included in the study based on the following criteria: (1) employed under the WfW program as a forestry worker; (2) responsible for mixing, handling, or spraying tasks; and (3) residing and working at one of the four study sites. Participants were selected through purposive sampling methods. Workers were not at risk of losing their employment through their participation in the study. The WfW management were aware and supportive that the scope of the study involved workers taking photographs of their work and home environment as part of strategies to improve their working conditions. 

### 2.3. Data Collection

Data for this study was collected through photovoice and a document review. 

#### 2.3.1. Photovoice 

This study used aspects of the novel community-based participatory research (CBPR) approach, photovoice, to document WfW forestry workers and their families’ herbicide take-home exposure risks. Photovoice uses visual methods to raise community concerns, address existing inequities, and ultimately drive social change [44,45,46,47]. Participants were issued cameras to photograph their life and work circumstances in relation to working with herbicides [47,48]. Through this visual representation, participants not only express concerns but more importantly, are able to influence decisions impacting their health. Photovoice has been instrumental in empowering marginalized communities to contribute to change within their communities [49]. This study omitted the “social action” pillar of photovoice research, in which study participants directly engage policy makers and officials about their concerns, because it was beyond the scope of the project. 

Each participant in the study was issued a disposable camera containing 27 exposures. They also received basic training on the use of a camera from fieldworkers who were conversant in Afrikaans and isiXhosa. The participants used the cameras to document their hygiene and other related practices for the duration of one week. The photos presented in this article are examples of the photos taken by participants. This was followed by four focus group discussions facilitated by the research staff and fieldworkers at all worksites, guided by the Photovoice Focus Group Guide. Each group discussion consisted of 10 WfW workers and lasted approximately 1.5 to 2 hours. The captured photographs were presented to the study participants, and a structured questionnaire consisting of general and specific questions was used to guide the discussions. This enabled participants to interpret the photographs, openly discuss their views, and critically engage on what they saw. The faces in the photographs were blurred to ensure and maintain the anonymity of the participants. All the focus group discussions were audio-recorded with the permission of the study participants and transcribed. 

#### 2.3.2. Document Review 

Eight WfW program operational and training documents were included in the study and assessed for information on take-home residue exposure risks. The eight WfW program documents that were assessed are as follows: (1) SANS 10118, 2011; (2) Policy on the use of herbicides and mycoherbicides for the control of alien vegetation, 2012; (3) Pesticide safety and application equipment: Sprayer operator pocket book, 2003; (4) First aiders and occupational health services facilities, 2012; (5) WfW workplace HIV/AIDS policy, 2004; (6) WfW rules and regulations, 2012; (7) WfW induction training manual; and 8) WfW invasive plant management–treatment methods, 2007. These cover workplace policies (e.g., WfW program HIV and AIDS policy), standard guidelines for contractors, guidelines for training and information, and educational material. In addition, three national and one international chemical policies were reviewed and assessed for provisions related to take-home exposure risks. 

### 2.4. Data Analysis

Data analysis comprised three phases, namely: (1) analysis of key WfW program documentation and national and international policies; (2) participatory analysis of photographs during focus group discussions; and (3) researchers’ analysis of all photographs captured by participants. Data from all three phases were imported into the qualitative data analysis software, Nvivo 11 (QSR International, Melbourne, Australia), and sorted, organized, and coded. 

The SA chemical legislation was specifically compared to the US legislation because this was the only legislation, to the knowledge of the researchers, which had provisions on take-home exposure risks. Additionally, most studies on take-home pesticide exposure risks were from the US, hence the comparison to the US legislation. Limited research exists on take-home pesticide exposure from other countries and regions. 

The second phase of data analysis was mainly driven by the study participants. They conveyed their views on the visual representation of their families being exposed to workplace residues at home and attempted to link this to underlying causes. The photographs and transcripts from the focus group discussions were coded using thematic analysis. This assisted in identifying recurring themes emerging from the data. In addition, the transcripts of all four focus group discussions were compared. Although the study would have benefitted from comparisons across the different worksites, this was beyond the scope of the study.

The third phase was an adaption of traditional photovoice methods and involved the analysis of the entire dataset of photographs captured by participants. This assisted with validating key themes identified by the participants and determining other underlying causes of take-home herbicide exposure risks not identified by participants.

## 3. Results and Discussion

This section presents the findings and discussion of the document review and photovoice.

### 3.1. Document Review Findings

#### 3.1.1. Absence of Take-Home Exposure in Workplace Policies

The absence of information on take-home residue exposure risks was identified as a key gap in the existing WfW program documentation and SA chemical legislation. The WfW Standard Operating Procedures (SOPs) primarily focused on the risks of herbicide use to the environment and workers’ health, in addition to the exposure reduction measures for workers at worksites. None of the reviewed WfW training documents addressed take-home exposure risks and the measures to reduce exposure. Furthermore, all information and education materials, such as the Pesticide safety and application equipment: Sprayer operator pocketbook 2003, which is issued to workers, omitted take-home exposure risks. Only one document (i.e., Policy on the use of herbicides and mycoherbicides for the control of alien vegetation, 2012) was specific to non-worker exposure, stating that those who live near treated areas should be informed of exposure risks. The remaining WfW program documentation that was reviewed (e.g., SANS 10118, 2011; First aiders and occupational health services facilities, 2012; and the WfW Invasive Plant Management–Treatment Methods, 2007) also excluded take-home exposure risks.

#### 3.1.2. Lack of Policy Support 

The prevention and management of occupational pesticide exposure in SA is guided by the Occupational Health and Safety Act (OHSA) (Act no.85 of 1993), the Regulations for Hazardous Chemical Agents 2021 (which repealed the recently active Hazardous Chemicals Regulations, 1995), and the Pesticide Management Policy for South Africa (2010). None of the abovementioned chemical legislation in SA address take-home exposure and potential health risks. The 2021 Regulations for Hazardous Chemical Agents refers to biological monitoring, including non-occupational exposures, but these are linked to neighborhood, food residue, and water and air borne exposures rather than occupational take home exposures. The US is currently guided by the Environmental Protection Agency (EPA) Agricultural Worker Protection Standard (WPS) (2015) and previously, the Workers’ Families Protection Act, 1992 (Table 2). The table below highlights the variance in the provisions on occupational exposure versus non-occupational exposure.

In both SA and the US, training is the standard approach used to inform workers of the risks of pesticides exposure. The US WPS (2015) requires employers to train workers on the risks of take-home exposure and the sources of exposure such as contaminated work clothing. The training should also encompass risk reduction practices for workers and their families to prevent and reduce exposure at home (e.g., washing and storing work clothes separately from household laundry and dry-cleaning PPE before storing). According to US WPS (2015), employers are further required to provide hygiene facilities at worksites for decontamination purposes. These include a gallon of water, soap, and single-use towels. On the contrary, the SA chemical legislation does not address take-home exposure risks and recommended practices for workers to reduce exposure risks, despite updating the chemical regulations in March 2021. Employers in SA are also not obliged to provide hygiene facilities at worksites.

The main difference between the SA and the US chemical legislation, therefore, is that the SA legislation falls short of recognizing take-home exposure risks for workers and their families. This may have contributed to the exclusion of take-home exposure risks in the WfW program documentation, and possibly influenced WfW workers practices at worksites and home. A survey conducted among farmers in five Midwestern States in the US found that government regulations and concerns for other family members motivated workers to adhere to workplace requirements [50]. This raises an important argument for workers in SA because they may not be aware of the risks of this type of exposure and take the necessary precautionary measures to protect their families. Therefore, incorporating provisions on take-home exposure in the national legislation and ensuring workplace policies are aligned is an important prevention measure for reducing take-home residue exposure risks. It is important to note that the absence of provisions on take-home exposure risks in the SA chemical legislation may be indicative of the lack of evidence that take-home exposure is a concern in the country. 

### 3.2. Photograph Findings

Three main themes related to take-home herbicide residue exposure risks emerged during the focus group discussions. These were: (1) workers’ workplace practices’ (2) workers’ after-work behaviours; and (3) home hygiene practices. Three sub-themes were also identified under the main theme of home-hygiene practices, namely, laundering practices, laundry drying practices, and storage practices. The identified themes correlated with those emerging from the analysis of the entire body of photographs. Through this researcher-led analysis, an additional theme, worker’s living conditions, was identified.

#### 3.2.1. Workplace Practices 

**Figure 2:**
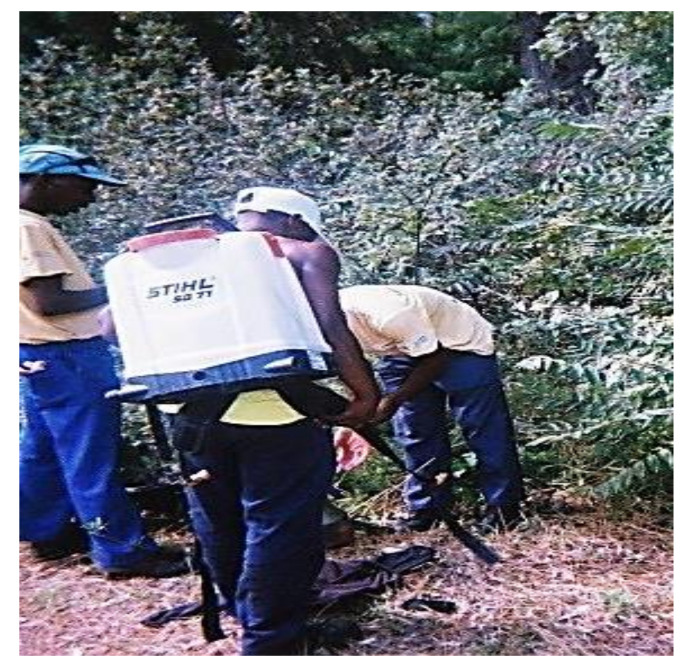
WfW worker’s dermal exposures to herbicide formulations and working conditions.

Several participants’ photographs revealed workers failing to implement the minimum precautionary measures, such as the use of gloves and long-sleeved t-shirts, while engaged in mixing and spraying tasks (Figure 2). This may have resulted in residues being transported into their homes through the dermal route. No workers wore the prescribed respirators while engaged in mixing, pouring, or handling tasks, and all workers were also exposed to residues through the respiratory route. Female workers were more likely to wear full PPE (i.e., trousers, long sleeved coat, safety boots, chemical resistant gloves, goggles, and a hard hat) than their male counterparts. An evaluation of a community-based participatory worksite intervention conducted in Lower Yakima Valley, Washington State, US, highlighted the importance of PPE in reducing the risk of exposure; the study found that workers who wore complete PPE had lower levels of dimethyl alkylphosphate (DMAP) and malathion dicarboxylic acid (MDA) concentrations in their urine [18]. Despite PPE being a protective measure against exposure associated with workers’ behaviors during handling and mixing tasks at worksites, the equipment may be a source of take-home exposure for their families, particularly when contaminated. Some photographs visually depicted the spillage of herbicide formulations at work and exposure risks; for example, one participant had blue herbicide dye spots on his PPE from carrying a back-sprayer applicator. Blue dye is mixed with herbicide formulations to provide a visual indication of the amount applied on plants, and to indicate which plants were treated. These findings were consistent with other studies that demonstrated the association between workers’ practices at work and the risk of exposure amongst their families [1,32,51]. Therefore, compliance with rules relating to wearing the required PPE at worksites remains an important factor in reducing take-home exposure risks. 

The working conditions of workers, such as forest terrain (Figure 2), was another factor which contributed to take-home exposure risks. Due to the transient nature of forestry work, hygiene facilities, mobile decontamination, and hygiene facilities (e.g., hand washing and changing facilities) were not available at the worksites, thus impacting workers’ ability to carry out hygiene practices at work. The absence of these facilities also resulted in workers taking their PPE home; this included contaminated PPE. In their study, Salvatore et al. (2008) noted that in addition to the training of workers, contextual and structural factors, such as the provision of hygiene facilities, strongly influenced whether they consistently implemented recommended practices [52]. Therefore, the provision of hygiene facilities at worksites will be a critical intervention in reducing the transportation of residues into workers’ homes. To accommodate the mobility of forestry work, mobile hygiene facilities may be more appropriate compared to fixed facilities.

#### 3.2.2. Post-Work Behaviours 

An additional theme that was identified by workers was post-work behaviours; that is, their care and maintenance of PPE when they arrive home after work. Just under half (i.e., 43%) of workers wore their PPE in the indoor environment and did not always change or shower immediately after work.

“*Some days it is too hot to wash your body right after work because one is just sweating too much*”.

(WfW worker A, Citrusdal)

These findings were similar to the results of two studies among agricultural workers in Lower Yakima Valley, Washington State, US, which reported that workers delayed showering immediately after work because they believed washing the body before it is cooled could cause pain in the bones and joints [19,53]. These studies highlighted the importance of workers’ perceptions and their social belief systems in determining whether they practice the recommended safety behaviours. Therefore, any interventions to reduce exposure risks should take these factors into account to enhance the effectiveness of recommended practices. 

**Figure 3:**
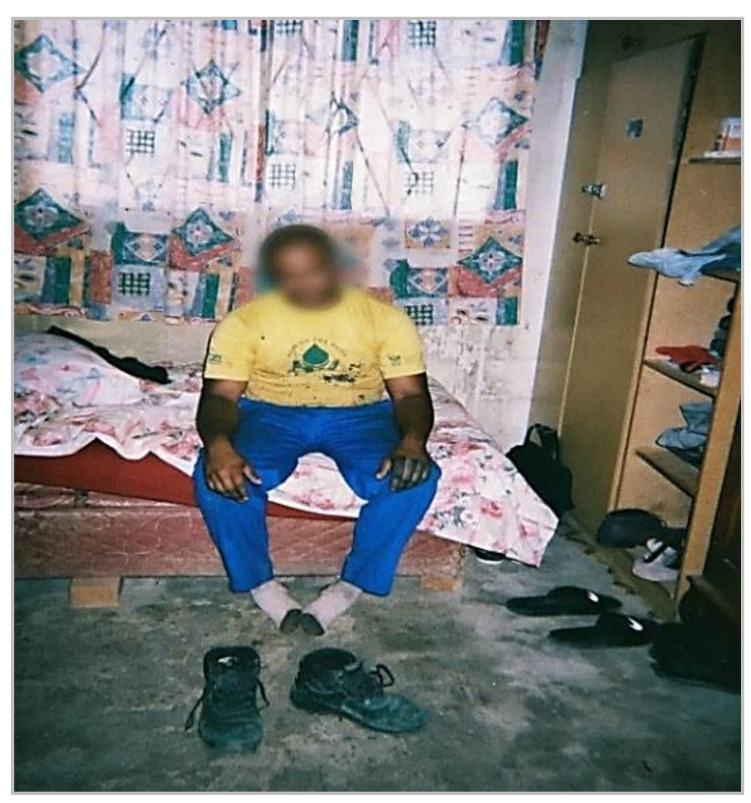
Herbicide residues (blue) on worker’s hands, clothing, and boots being removed at home.

Most workers entered their homes while wearing work boots soiled with herbicide dye. Only one participant removed their work boots outside the home; however, it was not clear whether this was a precautionary measure to reduce take-home exposure risks or a general practice of cleanliness. Other items of PPE (e.g., trousers and long sleeve coats) were often left out in the open on surfaces in the home (e.g., bed) that were used by other household members. Figure 3 provides evidence of worker’s transporting herbicide residues on their PPE into the home. One of the workers is sitting on a household surface wearing contaminated PPE, indicated by the blue dye (added to all WfW herbicides, as noted above) spots on the t-shirt. Blue spots were also noted on the worker’s arms and hands, indicative of direct exposure at worksites and potentially high residues. Therefore, the risk of contaminating household surfaces and exposing family members to residues is high. Other studies have documented similar findings, where most workers were reported to enter their homes while wearing work clothing [54,55,56]. 

The participants’ post-work behaviours were indicative of their lack of knowledge of take-home exposure risks and risk reduction measures. In their study among farmworkers in Salinas Valley, California, US, Cabrera and Leckie (2009) found that even when workers received training on general safety practices, the majority still wore their work boots inside the home [57]. This suggests that even when workers are well informed of take-home exposure risks, there are complexities in changing their behaviours at home. Therefore, addressing workers’ practices (e.g., mandatory changing, washing, or storing of PPE) at worksites may be more effective in reducing take-home herbicide exposure risks because control measures may be more easily enforced at work.

#### 3.2.3. Home Hygiene Practices

Home hygiene practices related to the care and maintenance of PPE was the third theme that emerged during the focus group discussions. Under this main theme, three sub-themes were identified, namely, laundering practices, laundry drying practices, and storage practices. 

##### Laundry Practices

**Figure 4:**
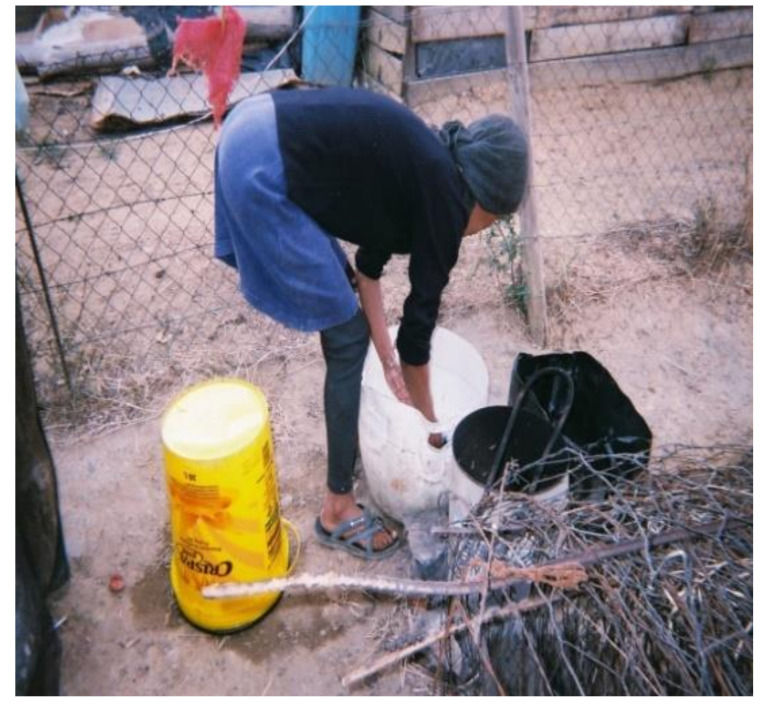
WfW worker handwashing PPE at home.

All WfW workers washed their PPE at home due to the absence of decontamination and washing facilities at worksites. Although all workers had access to piped water, the distance to these facilities varied. Some participants had running water inside their homes whereas others used communal water taps. The majority (i.e., 95%) of participants hand-washed their PPE (Figure 4) and only two used washing machines. Among those who hand-washed their PPE, none wore chemical resistant gloves to protect themselves from residues remaining on the work clothing. A study in Nebraska, US, also observed that most (i.e., 80%) applicators and launderers did not wear waterproof or chemical resistant gloves when laundering contaminated work clothing [34]. 

**Figure 5:**
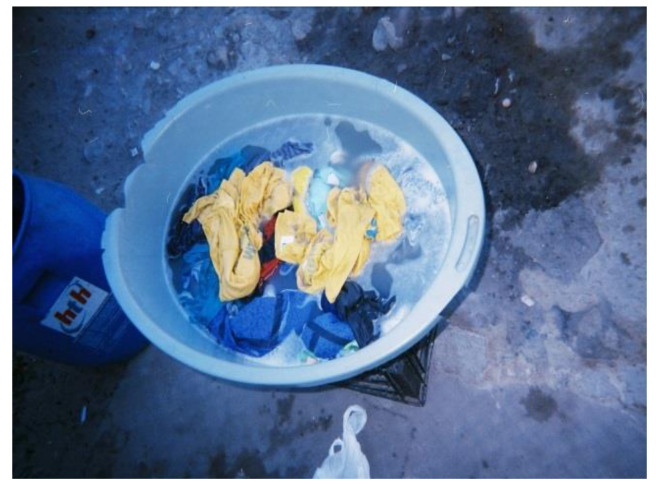
WfW workers’ PPE mixed with household laundry during washing.

Of the 37 participants in the study, 18 (49%) laundered their PPE separately from household laundry, whereas 4 (11%) participants mixed their PPE with household laundry (Figure 5). This was contrary to the findings reported in a study among farmworkers in Monterey County, California, US, where only six percent of workers washed their work clothes separately from household laundry [58]. The findings provide evidence that most of the workers appeared to be aware of the risks of mixing household laundry with contaminated PPE and employed protective measures to reduce the risks of exposure. It was still a concern, however, that some workers mixed household laundry with their PPE. During the focus group discussions, participants cited other factors which contributed to them not employing protectives measures while washing PPE. For example:

“*Washing PPE separately means taking many trips to fetch water and this is difficult as the water point is very far away*”.

(WfW worker G, Gouda)

**Figure 6:**
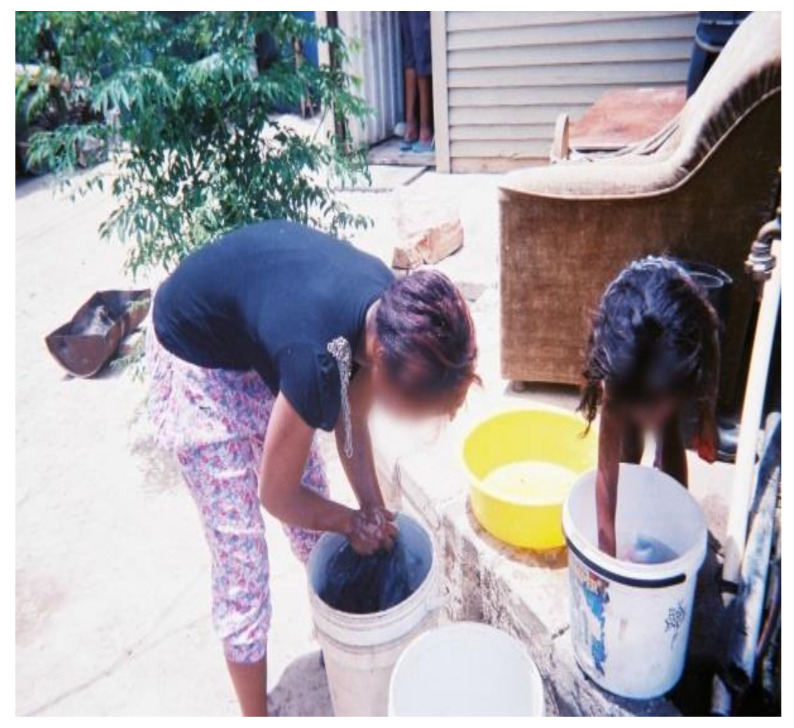
Child (right) touching herbicide-contaminated PPE water.

The water used to wash the herbicide-contaminated PPE was noted to be a source of potential exposures for children due to their behaviours such as assisting with washing (Figure 6). 

##### Laundry Drying Practices

**Figure 7:**
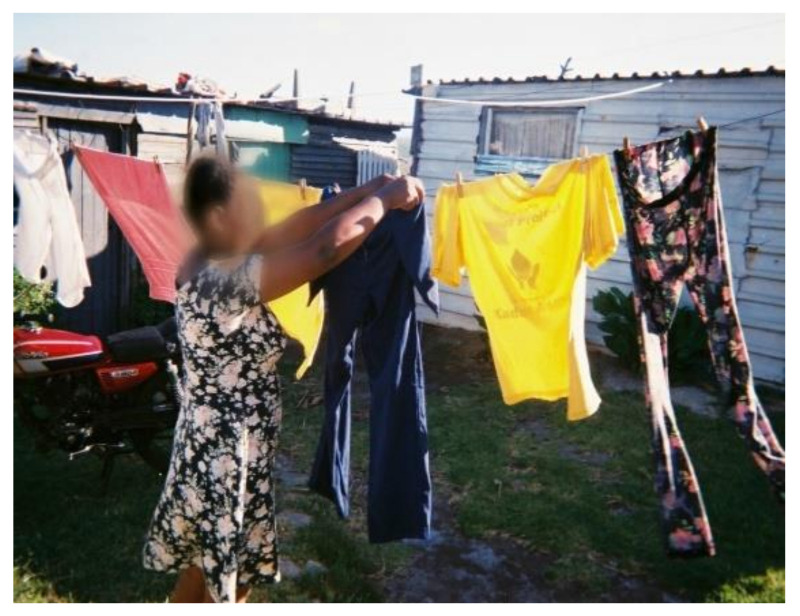
Washed PPE hung to dry with household laundry.

**Figure 8:**
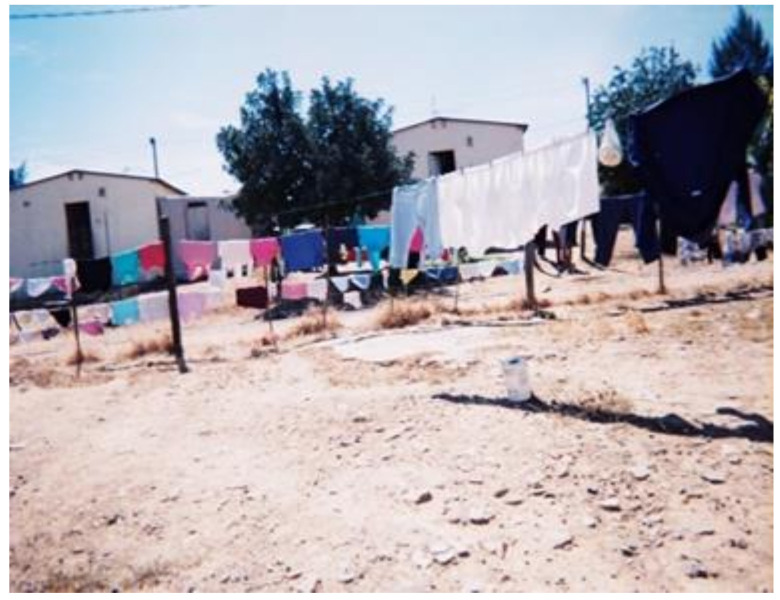
Children’s laundry hung separate from WfW PPE.

Practices related to drying washed PPE were inconsistent. Some workers dried their PPE together with household laundry (Figure 7) whereas others dried it separately (Figure 8). During the focus group discussions, participants held the view that drying PPE together with household laundry did not pose any risk, stating that they were unaware that residues could remain on washed PPE and be transferred to household laundry. 

##### Storage Practices

**Figure 9:**
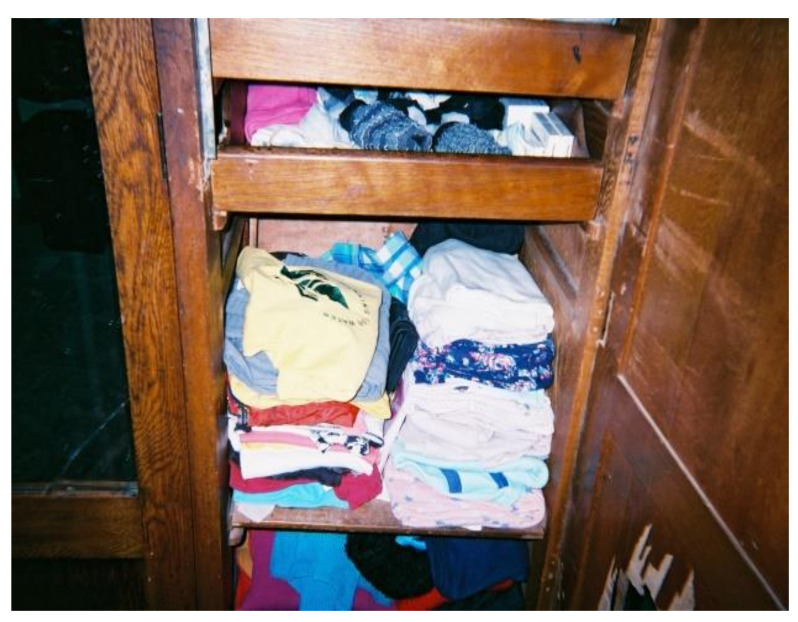
PPE stored with household laundry.

Although the majority of workers washed their PPE separately from household laundry, a similar trend was not observed in their storage practices. Most workers did not place their clean PPE in separate storage facilities from household laundry (Figure 9). Similar to the view held about drying practices, participants believed that the washed PPE no longer contained residues that could be transferred to household laundry as illustrated by the following comment:

“*PPE is placed in the cupboard after washing because the belief is that it has been cleaned and is free of herbicides*”.

(WfW worker D, Westlake)

There is minimal evidence in the existing literature of the effectiveness of home hygiene practices that translate to improved practices and reduce take-home exposure risks. A community-based intervention, the “For Healthy Kids” study in Eastern Washington State, US, found that the recommended practices in the US chemical legislation (e.g., removing work shoes and laundering work clothing separately from household laundry) were not associated with the reduction of urinary dimethylthiophospate concentrations in farmworkers and their children, in addition to azinphosmethyl concentrations in the house dust [59]. This inconclusive evidence supports the recommendation that interventions to prevent and reduce take-home exposure should be targeted at the source of exposure; that is, at worksites where exposure occurs. Although this may be more effective in reducing take-home exposure, it does not remove the responsibility of informing and educating workers’ families of this type of risk. Similar to the recommendations for workers’ families outlined in the US WPS (2015), WfW workers’ families should be informed of the following: that PPE may be contaminated with toxic herbicide residues, the associated health effects of herbicide exposure, precautions they may take to prevent and reduce exposure risks, decontamination processes for contaminated PPE, and low-risk cleaning practices. After receiving training, workers should be obliged to inform and provide educational material to their families on take-home exposure risks and precautionary measures they may take to reduce exposure. Further research is needed on effective methods of informing workers’ families of take-home exposure risks.

#### 3.2.4. Risk Promoting Living Conditions 

**Figure 10:**
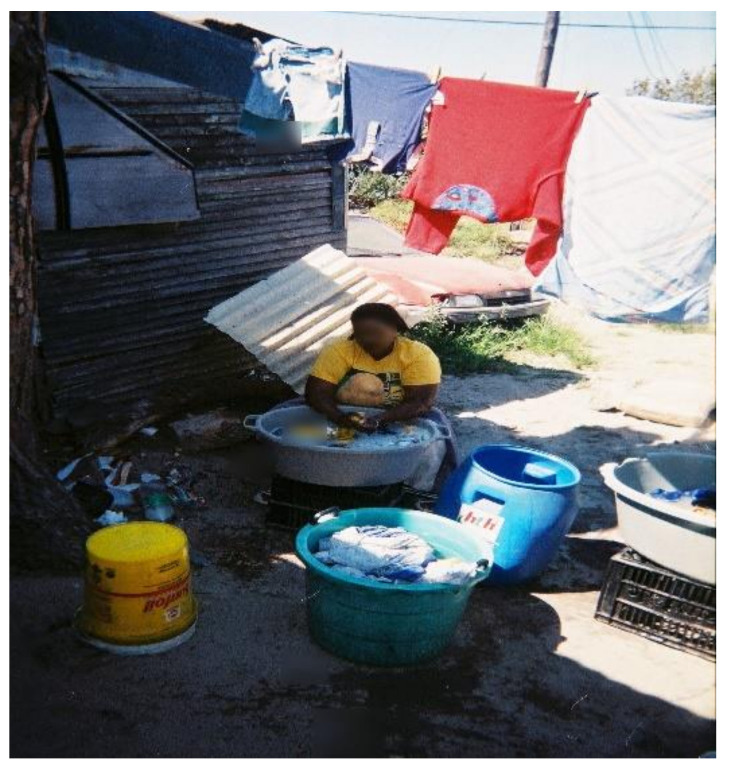
Worker’s living conditions.

A crucial theme that emerged from the photographs analyzed by the researchers was that of workers’ living conditions. WfW workers predominately live in townships that are characterized by informal (i.e., shacks) and low-cost housing (Figure 10). It was noted that the space within their homes was limited due to the size of the dwellings and overcrowding from multiple members living together. This may have contributed to their inability to employ protective practices such as storing and washing PPE separately from household laundry in order to reduce herbicide exposure risks. Arcury et al. (2009) observed that the homes of farmworkers had little space and, in most cases, were shared among many individuals. The study concludes that the farmers’ living conditions were not supportive or conducive for workers’ families to change their behaviours and practices [13]. 

**Figure 11:**
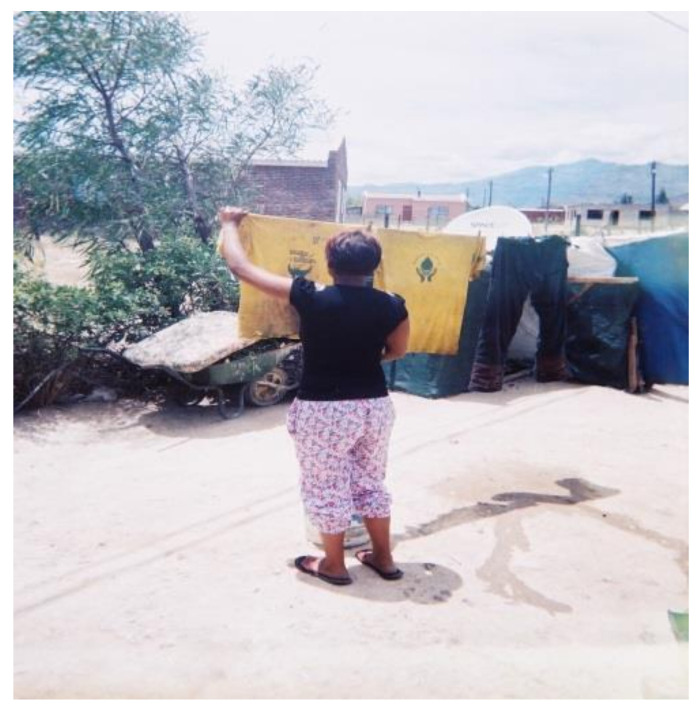
PPE remains contaminated even after handwashing.

It was also noted that because most WfW workers handwashed their PPE, they had difficulty in properly removing residues and soil from the PPE (Figure 11). Research is needed to assess whether herbicide residues remain on contaminated PPE and whether current washing practices at home are effective in removing residues. In addition, workers did not have proper drainage facilities in their homes to dispose of the contaminated water. For example, some workers threw the contaminated water on the ground in the yard of their home. Thus, workers’ living conditions are another important factor to consider in the design of interventions to reduce take-home herbicide exposure risks, because these conditions have the potential to hinder or enhance the effectiveness of interventions to reduce exposure risks.

“*We do not have drains or flush toilets so dirty water from washing PPE is thrown in refuse piles or holes around the home*”.

(WfW worker H, Citrusdal)

## 4. Study Limitations

The limitations of this study were as follows: (1) Photovoice is characterized by the involvement of study participants in different stages of the research process, including proposal development. Participants in this study did not contribute to the proposal development phase due to time constraints, which may have been a limitation in terms of incorporating their concerns as forestry workers. However, participants were involved in the process of data collection and data analysis, which gave them an opportunity to voice their concerns. (2) The short duration of the study contributed to the inability of fully incorporating the social action aspect. Participants did not present the photographs they captured revealing their families’ exposure risks to the WfW management for discussion. This engagement with the WfW management would have further empowered workers to contribute to the joint development of solutions that address take-home residue exposure risks. These limitations should be addressed in future research with a similar study design.

## 5. Conclusions

This study provided evidence of take-home herbicide residue exposure risks among South African WfW forestry workers and their families. The findings highlighted the need for integrated approaches to reduce take-home residue exposure risks for all workers applying herbicides. Given the exposure risks identified in the study and the gaps in the SA legislation to prevent these exposures, there are some lessons for improvement building on the protection of worker’s families legislation in the US. These include: (1) revising the existing SA chemical legislation to establish standards and regulations that specify measures for reducing take-home exposure risks from residues on work clothing; (2) providing mobile washing, changing, and storing facilities at worksites to target exposure at the source; (3) making provisions to dispose of contaminated PPE; (4) incorporating take-home exposure risks in workplace program documents (e.g., standard operating procedures and training material); (5) requiring training of workers on take-home exposure and ensuring they inform their families of this type of risk; and (6) providing instructions to workers on how they and their families can apply risk reduction practices (e.g., removing work boots and PPE before entering the home and washing or showering before coming into contact with family members, and providing instructions for those who clean PPE). 

To the knowledge of the authors, no other studies have assessed take-home exposure risks among forestry workers and their families in LMICs. Thus, replication studies are needed to appropriately characterize this problem in limited resource settings. Furthermore, take-home herbicide (and pesticide) residue exposure risks in LMICs should be explored using other evidenced-based research methods (e.g., biological and environmental sampling methods) to determine the extent of exposure among workers and their families. 

## Figures and Tables

**Table 1:** Study participant demographics by research site.

Research Site	Total Participants (*N* = 37)	Sex	Language
Citrusdal	11	Males = 5	Afrikaans
		Females = 6	
Gouda (de Hockestate)	10	Males = 4	English
		Females = 6	
Liesbeek River	8	Males = 1	Afrikaans (1) English (7)
		Females = 7	
Westlake	8	Males = 3	isiXhosa
		Females = 5	

**Table 2:** Comparison of SA and US legislative provisions for occupational and non-occupational exposure.

Chemical Legislation Provisions	United States	South Africa
Occupational Exposure (Protection of workers)	Environmental Protection Agency, Agricultural Worker Protection Standard (2015)	(1) Occupational Health and Safety Act (Act no.85 of 1993) (2) Hazardous Chemical Substances Regulations (1995)–recently replaced by the Regulations for Hazardous Chemical Agents (2021) (3) Pesticide management policy for South Africa (2010) formally Fertilizers, farm feeds, agricultural remedies and stock remedies Act (Act no.36 of 1947)
Non-occupational Exposure (Protection of workers’ families)	(1) Worker’s Families Protection Act (1992) (2) Environmental Protection Agency, Agricultural Worker Protection Standard (2015)	(1) None
